# Supplementary material for: Role of mHealth applications for emergency medical system activation in reducing mortality in low-income and middle-income countries: a systematic review protocol
Source: BMJ Open. 2022 Feb 23;12(2):e051792. doi: 10.1136/bmjopen-2021-051792 (PMC8867347; doi:10.1136/bmjopen-2021-051792)
Supplement: Author's manuscript [file bmjopen-2021-051792.draft_revisions.pdf]

BMJ Open

BMJ Open is committed to open peer review. As part of this commitment we make the peer review history of every article we publish publicly available.

When an article is published we post the peer reviewers' comments and the authors' responses online. We also post the versions of the paper that were used during peer review. These are the versions that the peer review comments apply to.

The versions of the paper that follow are the versions that were submitted during the peer review process. They are not the versions of record or the final published versions. They should not be cited or distributed as the published version of this manuscript.

BMJ Open is an open access journal and the full, final, typeset and author-corrected version of record of the manuscript is available on our site with no access controls, subscription charges or pay-per-view fees (http://bmjopen.bmj.com).

If you have any questions on BMJ Open's open peer review process please email info.bmjopen@bmj.com

BMJ Open

The role of mHealth applications for Emergency Medical System activation in reducing mortality in low-and middle-income countries: a systematic review protocol

Journal:	*BMJ Open*
Manuscript ID	bmjopen-2021-051792
Article Type:	Protocol
Date Submitted by the Author:	31-Mar-2021

Complete List of Authors:	Griswold, Dylan; Cambridge University, Clinical Neurosciences; Stanford University School of Medicine<br>Rubiano, Andres M; Università degli Studi del Piemonte Orientale Amedeo Avogadro Scuola di Medicina; Meditech Foundation

Keywords:	ACCIDENT & EMERGENCY MEDICINE, TRAUMA MANAGEMENT, Health policy < HEALTH SERVICES ADMINISTRATION & MANAGEMENT

SCHOLARONE™
Manuscripts

The role of mHealth applications for Emergency Medical System activation in reducing mortality in low-and middle-income countries: a systematic review protocol

Authors

Dylan P Griswold, BA[1,2,3] dg550@cam.ac.uk **Andres M. Rubiano, MD, PhD(c)**[1,4,5] andresrubiano@aol.com

1 NIHR Global Health Research Group on Neurotrauma, University of Cambridge, Cambridge, UK 2 Division of Neurosurgery, Department of Clinical Neurosciences, Addenbrooke's Hospital & University of Cambridge, Cambridge, UK 3 Stanford University School of Medicine, Stanford, CA, USA 4 Università del Piemonte Orientale (UPO) and Vrije Universiteit Brussel (VUB). 5 Meditech Foundation, Cali, Colombia.

Correspondence:

Andres M. Rubiano

Neuroscience Institute, INUB-MEDITECH Research Group

El Bosque University

Bogotá, Colombia

andresrubiano@aol.com

+57 300 6154775

review only

Abstract

Introduction: Prehospital care is an essential component in reducing mortality for patients presenting with emergency medical conditions. Since 90% of trauma related deaths occur in low-and middle-income countries, the importance of prehospital systems is even more critical. Prehospital systems tend to be underdeveloped or non-existent in these areas, with less than 1% of LMIC populations served by an organized prehospital system. Mobile health apps for activation of Emergency Medical System (EMS) have been shown to decrease mortality, but there has yet to be a systematic review and meta-analysis performed to clarify the role that these apps play in reducing mortality in LMICs. The objective of this review is to evaluate the effectiveness of mobile health apps for EMS activation versus traditional EMS dispatches in survival and transport time in patients with emergency medical conditions.

Methods and Analysis: The proposed systematic review of randomized controlled trials (RCTs) and non-randomized controlled trials (NRCTs) will be conducted in accordance with the Joanna Briggs Institute methodology for systematic reviews of effectiveness evidence. MEDLINE, CINAHL, Web of Science, Cochrane Library, EMBASE, and EBSCO will be searched from January 2005 to March 2021. The search results will be presented according to the Preferred Reporting Items for Systematic Reviews and Meta-Analyses (PRISMA) flow diagram. Primary outcomes will include mortality and transport time. Critical appraisal will be assessed using the JBI SUMARI Risk-of-Bias Tool for RCTs, and Risk-of-Bias In Non-Randomized Studies tool for NRCTs. A narrative synthesis will be conducted for all included studies. If sufficient data are available, a meta-analysis will be conducted. I^2 statistics will be used to assess heterogeneity and identify their potential sources.

Ethics and Dissemination: No ethical approval will be required, as this review is based on already published data and does not involve interaction with human subjects. The plan for dissemination, however, is to publish the findings of the review in a peer-reviewed journal and present findings at high- level international conferences that engage the most pertinent stakeholders. Any amendments to this protocol will be documented in the final review.

For peer review only

ARTICLE SUMMARY

Strengths and Limitations of this study

- To the best of our knowledge this protocol provides a detailed description of the first systematic review on the effectiveness of mobile health apps for EMS activations in reducing mortality in low-and middle-income countries
- The protocol adheres to the Preferred Reporting Items for Systematic Reviews and Meta-Analyses Protocols guidelines for reporting a systematic review protocol
- Given the niche nature of the topic it is possible there may not be enough data to perform a meta-analysis

For peer review only

Introduction

Prehospital care is an essential component in reducing mortality for patients presenting with emergency medical conditions. Given that 90% of trauma related deaths occur in low-and middle-income countries, the importance of prehospital systems is essential.[1] Prehospital systems tend to be underdeveloped or non-existent in these areas, with less than 1% of low-and middle-income country (LMIC) populations served by an organized prehospital system.[2] Mobile health apps for Emergency Medical Systems (EMS) activation have been shown to decrease mortality, but EMS development in many LMICs is in its infancy and remains underdeveloped compared to the already existing need for better prehospital care and transport. [3–6] A literature review evaluating the status of EMS in 16 LMICs found that most of the included countries lacked an organized prehospital system and were greatly hindered by financial constraints.[7] Another constraint was found to be a lack of the importance of an EMS among locals. The review found that countries with well-developed prehospital care like the US and the UK had the most influence in the development of well-functioning EMS through the implementation of training programs, support of local EMS leadership, advocacy and awareness, and funding.

Mobile health (mHealth) services hold promises in helping bridge the gap between the organization of a functional EMS and a well-functioning prehospital system. There is a wide range of cheap, easily accessible tools which can be used at the point of dispatch or care delivery. With a rapid increase in cellular services, health workers have new opportunities to reach and treat patients that they could not before. There has been an increase in the number of mHealth projects currently active in LMICs, but these tend to be pilot studies that have not been scaled up.[8] The good news is that with COVID19 came a surge in implementation of mHealth strategies to improve pandemic management and response. Countries in which these strategies were used for planning, surveillance, testing, contact tracing, quarantine, and healthcare were more successful.[9] Countries that can leverage this existing network to improve their EMS will likely find a noticeable improvement in prehospital care soon.

While it is unlikely there is a large body of studies examining the effect of mHealth apps for EMS activation regarding mortality and transport time in LMIC populations, it is important to gain a better understanding of the studies that have been published and for which there is data worth appraising. There has yet to be a systematic review and meta-analysis performed to clarify the role mHealth apps for EMS activation play in reducing mortality in LMICs, and our objective is to carry this out to provide data that will help improve mHealth apps for EMS dispatch in LMICs.

Methods

A protocol of this systematic review following the PRISMA statement was registered in the International Prospective Registry of Systematic Reviews. Any changes to the protocol will be amended in PROSPERO and reported in the final review. This systematic review was conducted following the JBI methodology for systematic reviews and meta-analyses.[10] This protocol adheres to the Preferred Reporting Items for Systematic Reviews and Meta-Analyses protocols (PRISMA-P) 2015.[11]

Patient and public involvement

Patients and the public were not involved in the design of this systemic review protocol.

For peer review only

For peer review only - http://bmjopen.bmj.com/site/about/guidelines.xhtml

Study design

A systematic review of peer review literature following the Preferred Reporting Items for Systematic Reviews and Meta-Analyses (PRISMA) approach by Moher et al is planned for this review.[11] Figure 1 summarises the planned stages of the review as described in this protocol.

Data source and search strategy

The search strategy will aim to locate both published and unpublished studies, including the grey literature. An initial limited search of MEDLINE (via PubMed) and CINAHL was undertaken to identify articles on the topic. The text words contained in the titles and abstracts of relevant articles, and the index terms used to describe the articles will be used to develop a full search strategy for Web of Science, Cochrane Library, EMBASE, and CINAL (EBSCO) will be searched from January 2005 to March 2021 to reflect contemporary practice *(see Appendix 1*). There will be no restriction regarding the language. The search strategy, including all identified keywords and index terms, will be adapted for each included database and/or information source. The reference list of all included sources of evidence will be screened for additional studies. EndNote20 will be used to store, organize, and manage all references

The existence of controlled descriptors (MeSH terms and CINAHL headings) and their key words was verified in each database. The search terms were combined using the Boolean operators 'AND' and 'OR.'[12]

A search strategy combining MeSH terms and free-text words such as –(mobile OR telemedicine* OR ehealth OR telehealth OR smartphone* OR "smart phone*") AND ("emergency medical service*" OR "emergency medical system*" OR "emergency health service*" OR EMS) AND Cochrane's LMIC search filter.[13]

Selection of studies

Following the search, all identified citations will be collated and uploaded into EndNote20 (Clarivate Analytics, PA, USA). The citations will then be imported into JBI SUMARI for the review process. Two independent reviewers will examine titles and abstracts for eligibility. The full text of selected studies will be retrieved and assessed. Full-text studies that do not meet the inclusion criteria will be excluded, and a list of such excluded studies will be provided. Disagreements between the reviewers during title and abstract screening or full-text screening, will be resolved by consensus, or with a third reviewer. The results of the search will be reported in full in the final report and presented in a Preferred Reporting Items for Systematic Reviews and Meta-analyses (PRISMA) flow diagram.

Eligibility criteria

Inclusion criteria

Participants

This review will include patients of any age with emergency medical conditions including but not limited to stroke, myocardial infarction, trauma, and respiratory failure.

For peer review only

Intervention(s)

Mobile health EMS dispatch, alerts, triage systems and applications

Comparator(s)

Traditional EMS dispatch, alerts, and triage systems.

Outcomes

This review will consider studies that include the following outcomes: mortality and transport time expressed as incidence differences, risk ratios or odds ratios. All outcomes will be summarized narratively.

Types of studies

This review will consider both experimental and quasi-experimental study designs including randomized controlled trials, non-randomized controlled trials, before and after studies and interrupted time-series studies. In addition, analytical observational studies including prospective and retrospective cohort studies, case-control studies and analytical cross-sectional studies will be considered for inclusion. This review will also consider descriptive observational study designs including case series, individual case reports and descriptive cross-sectional studies for inclusion. We will include preprint studies identified in our search, but no ongoing studies will be considered.

Assessment of methodological quality

Eligible studies will be critically appraised by two independent reviewers at the study level using standardized critical appraisal instruments from the Joanna Briggs Institute for experimental and quasi-experimental studies. Authors of papers will be contacted to request missing or additional data for clarification, where required. Any disagreements that arise will be resolved through discussion, or with a third reviewer. The results of critical appraisal will be reported in narrative form and in a table.

All studies, regardless of the results of their methodological quality, will undergo data extraction and synthesis (where possible).

Data extraction

Data will be extracted from studies included in the review by two independent reviewers using the standardized data extraction tool. The data extracted will include specific details about the populations, study methods, interventions, and outcomes of significance to the review objective. Any disagreements that arise between the reviewers will be resolved through discussion, or with a third reviewer. Authors of papers will be contacted to request missing or additional data, where required.

Data synthesis

Studies will, where possible be pooled in statistical meta-analysis using JBI SUMARI. Effect sizes will be expressed as either odds ratios (for dichotomous data) and weighted (or standardized) final post-intervention mean differences (for continuous data) and their 95% confidence intervals will be

For peer review only - http://bmjopen.bmj.com/site/about/guidelines.xhtml

calculated for analysis. Heterogeneity will be assessed statistically using the standard chi squared and I^2 tests. Statistical analyses will be performed using a fixed effect model.[14] Sensitivity analyses will also be conducted. Where statistical pooling is not possible the findings will be presented in narrative form including tables and figures to aid in data presentation where appropriate. A funnel plot will be generated in Microsoft Excel (Seattle, WA) to use to assess publication bias if there are 10 or more studies included in a meta-analysis. Statistical tests for funnel plot asymmetry (Egger test, Begg test, Harbord test) will be performed where appropriate.

Assessing certainty in the findings

The Grading of Recommendations, Assessment, Development and Evaluation (GRADE) approach for grading the certainty of evidence will be followed.[15] The SoF will present the following information where appropriate: absolute risks for the treatment and control, estimates of relative risk, and quality of the evidence based on the risk of bias, directness, heterogeneity, precision, and risk of publication bias. The outcomes reported in the SoF will be mortality and transport time.

Ethics and dissemination

No ethical approval will be required, as this review is based on already published data and does not involve interaction with human subjects. The plan for dissemination, however, is to publish the findings of the review in a peer-reviewed journal and present findings at high- level international conferences that engage the most pertinent stakeholders. Any amendments to this protocol will be documented in the final review.

DISCUSSION

This protocol has been rigorously developed and designed specifically to assess the effects of mHealth apps for EMS activation on mortality and transport time in LMICs. Given the likely limited evidence associated with the primary objective, findings from the review will be critical for researchers, policymakers, government, and non-governmental organisations for planning, developing, and improving mHealth apps for EMS activation in LMICs. If protocol modifications are required, the authors will include the detailed description of any changes along with a justification during the publication of the review.

The main barriers for implementing mHealth apps for EMS activation include regulatory bodies, technological expertise, and user interaction. Usability of the application, signal loss, data volume utilization, need to enter passwords, and the availability of automated or in-app context-relevant clinical advice are important considerations for the three groups involved: experts, front-line healthcare workers, and patients. This review will serve as an important role as a repository of available evidence for the purpose of addressing these barriers and setting effective policy and guideline recommendations.

For peer review only

Contributors AMR conceived the review. DG wrote an initial draft of the manuscript. DG and AMR refined the review design. Both authors were involved in subsequent draft manuscript reviews and updates and approved the final version of this protocol.

Funding Mr Griswold was supported by the Gates Cambridge (Grant #: OPP1144) and Dr Rubiano was supported by the CRIMEDIM center at Università del Piemonte Orientale (UPO) (Grant #: N/A).

Competing interests None declared.

Patient consent for publication Not required.

Data statement: This review is based on previously published data. Any relevant data will be published with the review as either an appendix or as an online supplement.

Acknowledgements: This article is the result of a study conducted in the framework of the International PhD in Global Health, Humanitarian Aid and Disaster Medicine jointly organized by Università del Piemonte Orientale (UPO) and Vrije Universiteit Brussel (VUB). The authors would like to thank Stanford University research librarian, Lily Ren for her invaluable contribution in helping to develop a relevant and robust search strategy.

Open access This is an open access article distributed in accordance with the Creative Commons Attribution Non-Commercial (CC BY-NC 4.0) license, which permits others to distribute, remix, adapt, build upon this work non-commercially, and license their derivative works on different terms, provided the original work is properly cited, appropriate credit is given, any changes made indicated, and the use is non-commercial. See: http://creativecommons.org/licenses/by-nc/4.0/.

License to BMJ Group for Publication: I ***Andres M. Rubiano*** The Corresponding Author of this article contained within the original manuscript which includes any diagrams & photographs within and any related or stand-alone film submitted (the Contribution") has the right to grant on behalf of all authors and does grant on behalf of all authors, a license to the BMJ Publishing Group Ltd and its licensees, to permit this Contribution (if accepted) to be published in the BMJ and any other BMJ Group products and to exploit all subsidiary rights, as set out in our license set out at: http://www.bmj.com/about-bmj/resources-authors/forms-policies-and-checklists/copyright-open-access-and-permission-reuse.

For peer review only

Appendices

Appendix I: MEDLINE via PubMed search strategy

Search conducted on 26 March 2021

Search	Query	Results
#12	Search: **#11 AND #10** Sort by: **Most Recent**	397
#11	Search: **("Smartphone"[Mesh] OR "Telemedicine"[Mesh] OR "Mobile Applications"[Mesh] OR "mobile"[tw] OR "ehealth"[tw] OR "telehealth"[tw] OR "telemedicine"[tw] OR "smartphone*"[tw] OR "smart phone*"[tw]) AND ("Emergency Medical Services"[Mesh] OR "emergency medical service*"[tw] OR "emergency medical system*"[tw] OR "emergency health service*"[tw] OR EMS[tw])** Sort by: **Most Recent**	4,072
#10	Search: **afghanistan[MeSH] OR albania[MeSH] OR algeria[MeSH] OR american samoa[MeSH] OR angola[MeSH] OR antigua and barbuda[MeSH] OR argentina[MeSH] OR armenia[MeSH] OR aruba[MeSH] OR azerbaijan[MeSH] OR bahrain[MeSH] OR bangladesh[MeSH] OR barbados[MeSH] OR republic of belarus[MeSH] OR belize[MeSH] OR benin[MeSH] OR bhutan[MeSH] OR bolivia[MeSH] OR bosnia and herzegovina[MeSH] OR botswana[MeSH] OR brazil[MeSH] OR bulgaria[MeSH] OR burkina faso[MeSH] OR burundi[MeSH] OR cabo verde[MeSH] OR cambodia[MeSH] OR cameroon[MeSH] OR central african republic[MeSH] OR chad[MeSH] OR chile[MeSH] OR china[MeSH] OR colombia[MeSH] OR comoros[MeSH] OR democratic republic of the congo[MeSH] OR congo[MeSH] OR costa rica[MeSH] OR cote d'ivoire[MeSH] OR croatia[MeSH] OR cuba[MeSH] OR cyprus[MeSH] OR czech republic[MeSH] OR djibouti[MeSH] OR dominica[MeSH] OR dominican republic[MeSH] OR ecuador[MeSH] OR egypt[MeSH] OR el salvador[MeSH] OR equatorial guinea[MeSH] OR eritrea[MeSH] OR estonia[MeSH] OR swaziland[MeSH] OR ethiopia[MeSH] OR fiji[MeSH] OR gabon[MeSH] OR gambia[MeSH] OR georgia (republic)[MeSH] OR ghana[MeSH] OR gibraltar[MeSH] OR greece[MeSH] OR grenada[MeSH] OR guam[MeSH] OR guatemala[MeSH] OR guinea[MeSH] OR guinea bissau[MeSH] OR guyana[MeSH] OR haiti[MeSH] OR honduras[MeSH] OR hungary[MeSH] OR india[MeSH] OR indonesia[MeSH] OR iran[MeSH] OR iraq[MeSH]**	

Search	Query	Results
	OR jamaica[MeSH] OR jordan[MeSH] OR kazakhstan[MeSH] OR kenya[MeSH] OR democratic people's republic of korea[MeSH] OR republic of korea[MeSH] OR kosovo[MeSH] OR kyrgyzstan[MeSH] OR laos[MeSH] OR latvia[MeSH] OR lebanon[MeSH] OR lesotho[MeSH] OR liberia[MeSH] OR libya[MeSH] OR lithuania[MeSH] OR macau[MeSH] OR republic of north macedonia[MeSH] OR madagascar[MeSH] OR malawi[MeSH] OR malaysia[MeSH] OR indian ocean islands[MeSH] OR mali[MeSH] OR malta[MeSH] OR micronesia[MeSH] OR palau[MeSH] OR mauritania[MeSH] OR mauritius[MeSH] OR mexico[MeSH] OR moldova[MeSH] OR mongolia[MeSH] OR montenegro[MeSH] OR morocco[MeSH] OR mozambique[MeSH] OR myanmar[MeSH] OR namibia[MeSH] OR nepal[MeSH] OR netherlands antilles[MeSH] OR nicaragua[MeSH] OR niger[MeSH] OR nigeria[MeSH] OR oman[MeSH] OR pakistan[MeSH] OR panama[MeSH] OR papua new guinea[MeSH] OR paraguay[MeSH] OR peru[MeSH] OR philippines[MeSH] OR poland[MeSH] OR portugal[MeSH] OR puerto rico[MeSH] OR romania[MeSH] OR russia[MeSH] OR rwanda[MeSH] OR samoa[MeSH] OR sao tome and principe[MeSH] OR saudi arabia[MeSH] OR senegal[MeSH] OR serbia[MeSH] OR seychelles[MeSH] OR sierra leone[MeSH] OR slovakia[MeSH] OR slovenia[MeSH] OR melanesia[MeSH] OR somalia[MeSH] OR south africa[MeSH] OR south sudan[MeSH] OR sri lanka[MeSH] OR saint kitts and nevis[MeSH] OR saint lucia[MeSH] OR saint vincent and the grenadines[MeSH] OR sudan[MeSH] OR suriname[MeSH] OR syria[MeSH] OR tajikistan[MeSH] OR tanzania[MeSH] OR thailand[MeSH] OR timor leste[MeSH] OR togo[MeSH] OR tonga[MeSH] OR trinidad and tobago[MeSH] OR tunisia[MeSH] OR turkey[MeSH] OR turkmenistan[MeSH] OR uganda[MeSH] OR ukraine[MeSH] OR uruguay[MeSH] OR uzbekistan[MeSH] OR vanuatu[MeSH] OR venezuela[MeSH] OR vietnam[MeSH] OR middle east[MeSH] OR yemen[MeSH] OR yugoslavia[MeSH] OR zambia[MeSH] OR zimbabwe[MeSH] OR africa south of the sahara[MeSH] OR africa, central[MeSH] OR africa, northern[MeSH] OR africa, southern[MeSH] OR africa, eastern[MeSH] OR africa, western[MeSH] OR west indies[MeSH] OR indian ocean islands[MeSH] OR caribbean region[MeSH] OR central america[MeSH] OR latin america[MeSH] OR south america[MeSH] OR asia, central[MeSH] OR asia, northern[MeSH] OR asia, southeastern[MeSH] OR asia, western[MeSH] OR europe,	

Search	Query	Results
	**eastern[MeSH] OR developing countries[MeSH]** Sort by: **Most Recent**	

References

1. Hofman K, Primack A, Keusch G, Hrynkow S. Addressing the growing burden of trauma and injury in low- and middle-income countries. *Am J Public Health*. 2005;95(1):13–7.

2. Nielsen K, Mock C, Joshipura M, Rubiano AM, Zakariah A, Rivara F. Assessment of the Status of Prehospital Care in 13 Low- and Middle-Income Countries. *Prehosp Emerg Care*. 2012;16(3):381–9.

3. Mbugua P, Otieno C, Kayima J, Amayo A, McLigeyo S. Diabetic ketoacidosis: clinical presentation and precipitating factors at Kenyatta National Hospital, Nairobi. *E Afr Med J*. 2006;82(12):S191-6.

4. Gaieski DF, Goyal M. History and current trends in sudden cardiac arrest and resuscitation in adults. *Hosp Pract 1995*. 2010;38(4):44–53.

5. Terkelsen CJ, Sørensen JT, Maeng M, Jensen LO, Tilsted H-H, Trautner S, Vach W, Johnsen SP, Thuesen L, Lassen JF. System Delay and Mortality Among Patients With STEMI Treated With Primary Percutaneous Coronary Intervention. *Jama*. 2010;304(7):763.

6. Siman-Tov M, Radomislensky I, ITG, Peleg K. Reduction in trauma mortality in Israel during the last decade (2000–2010): The impact of changes in the trauma system. *Inj*. 2013;44(11):1448–52.

7. Suryanto, Plummer V, Boyle M. EMS Systems in Lower-Middle Income Countries: A Literature Review. *Prehospital Disaster Medicine*. 2016;32(1):64–70.

8. Wallis L, Blessing P, Dalwai M, Shin SD. Integrating mHealth at point of care in low- and middle-income settings: the system perspective. *Global Health Action*. 2017;10(sup3):1327686.

9. Whitelaw S, Mamas MA, Topol E, Spall HGCV. Applications of digital technology in COVID-19 pandemic planning and response. *Lancet Digital Heal*. 2020;2(8):e435–40.

10. Aromataris E, Munn Z, Tufanaru C, Campbell J, Hopp L. JBI Reviewer's Manual. 2019;

11. Moher D, Liberati A, Tetzlaff J, Altman DG, Group P. Preferred reporting items for systematic reviews and meta-analyses: the PRISMA statement. *International journal of surgery (London, England)*. 2010;8:336–41.

12. Lefebvre C, Manheimer E, Glanville J. Cochrane Handbook for Systematic Reviews of Interventions. 2008;95–150.

For peer review only

For peer review only - http://bmjopen.bmj.com/site/about/guidelines.xhtml

13. Cochrane, Library W, Collaboration C. Cochrane LMIC Filters [Internet]. undefined. 2020 [cited 2021 Mar 26]. Available from: https://epoc.cochrane.org/lmic-filters

14. Tufanaru C, Munn Z, Stephenson M, Aromataris E. Fixed or random effects meta-analysis? Common methodological issues in systematic reviews of effectiveness. *Int J Evidence-based Healthc*. 2015;13(3):196–207.

15. Grading quality of evidence and strength of recommendations. *Bmj*. 2004;328(7454):1490.

Figure 1. Summary of search strategy process

For peer review only

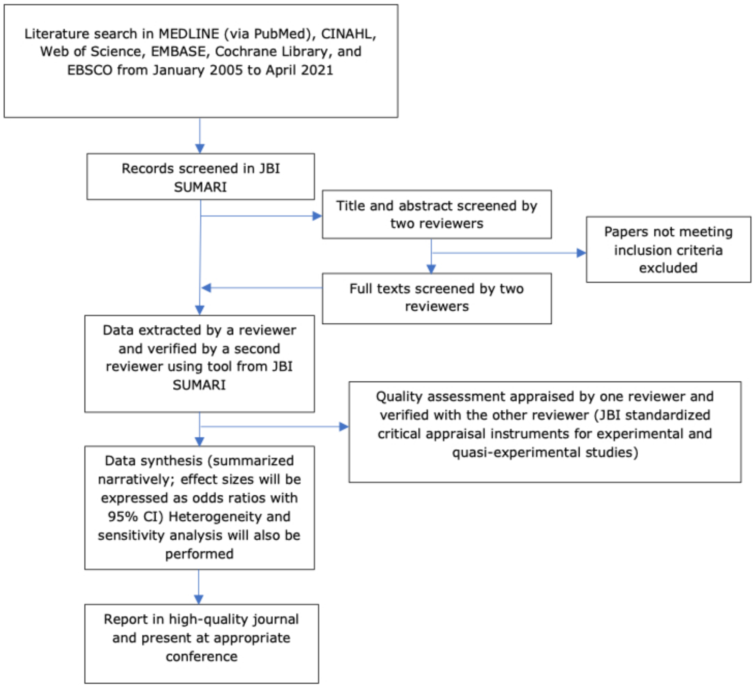

Figure 1 Summary of search strategy search process.

Figure 1. Summary of search strategy process

340x288mm (72 x 72 DPI)

PRISMA-P 2015 Checklist

This checklist has been adapted for use with protocol submissions to *Systematic Reviews* from Table 3 in Moher D et al: Preferred reporting items for systematic review and meta-analysis protocols (PRISMA-P) 2015 statement. *Systematic Reviews* 2015 **4**:1

Section/topic	#	Checklist item	Information reported: Yes	Information reported: No	Line number(s)
**ADMINISTRATIVE INFORMATION**					
**Title**					
Identification	1a	Identify the report as a protocol of a systematic review	☒	☐	2,3
Update	1b	If the protocol is for an update of a previous systematic review, identify as such	☐	☒	
**Registration**	2	If registered, provide the name of the registry (e.g., PROSPERO) and registration number in the Abstract	☐	☒	
**Authors**					
Contact	3a	Provide name, institutional affiliation, and e-mail address of all protocol authors; provide physical mailing address of corresponding author	☒	☐	5-9
Contributions	3b	Describe contributions of protocol authors and identify the guarantor of the review	☒	☐	193-195
**Amendments**	4	If the protocol represents an amendment of a previously completed or published protocol, identify as such and list changes; otherwise, state plan for documenting important protocol amendments	☐	☒	
**Support**					
Sources	5a	Indicate sources of financial or other support for the review	☒	☐	196-197
Sponsor	5b	Provide name for the review funder and/or sponsor	☒	☐	196-197
Role of sponsor/funder	5c	Describe roles of funder(s), sponsor(s), and/or institution(s), if any, in developing the protocol	☒	☐	196-197
**INTRODUCTION**					
**Rationale**	6	Describe the rationale for the review in the context of what is already known	☒	☐	45-68
**Objectives**	7	Provide an explicit statement of the question(s) the review will address with reference to participants, interventions, comparators, and outcomes (PICO)	☒	☐	69-74
**METHODS**					

For peer review only

Section/topic	#	Checklist item	Information reported: Yes	Information reported: No	Line number(s)
**Eligibility criteria**	8	Specify the study characteristics (e.g., PICO, study design, setting, time frame) and report characteristics (e.g., years considered, language, publication status) to be used as criteria for eligibility for the review	☒	☐	115-135
**Information sources**	9	Describe all intended information sources (e.g., electronic databases, contact with study authors, trial registers, or other grey literature sources) with planned dates of coverage	☒	☐	89-98
**Search strategy**	10	Present draft of search strategy to be used for at least one electronic database, including planned limits, such that it could be repeated	☒	☐	234…
STUDY RECORDS					
Data management	11a	Describe the mechanism(s) that will be used to manage records and data throughout the review	☒	☐	107-108
Selection process	11b	State the process that will be used for selecting studies (e.g., two independent reviewers) through each phase of the review (i.e., screening, eligibility, and inclusion in meta-analysis)	☒	☐	109-114
Data collection process	11c	Describe planned method of extracting data from reports (e.g., piloting forms, done independently, in duplicate), any processes for obtaining and confirming data from investigators	☒	☐	145-149
**Data items**	12	List and define all variables for which data will be sought (e.g., PICO items, funding sources), any pre-planned data assumptions and simplifications	☐	☒	
**Outcomes and prioritization**	13	List and define all outcomes for which data will be sought, including prioritization of main and additional outcomes, with rationale	☒	☐	146-149
**Risk of bias in individual studies**	14	Describe anticipated methods for assessing risk of bias of individual studies, including whether this will be done at the outcome or study level, or both; state how this information will be used in data synthesis	☒	☐	137-143
DATA					
**Synthesis**	15a	Describe criteria under which study data will be quantitatively synthesized	☐	☒	
	15b	If data are appropriate for quantitative synthesis, describe planned summary measures, methods of handling data, and methods of combining data from studies, including any planned exploration of consistency (e.g., I^2, Kendall's tau)	☒	☐	151-155
	15c	Describe any proposed additional analyses (e.g., sensitivity or subgroup analyses, meta-regression)	☒	☐	155-157
	15d	If quantitative synthesis is not appropriate, describe the type of summary planned	☐	☒	
**Meta-bias(es)**	16	Specify any planned assessment of meta-bias(es) (e.g., publication bias across studies, selective reporting within studies)	☒	☐	157-160
**Confidence in cumulative evidence**	17	Describe how the strength of the body of evidence will be assessed (e.g., GRADE)	☒	☐	162-166

For peer review only

BioMed Central The Open Access Publisher

BMJ Open

The role of mHealth applications for Emergency Medical System activation in reducing mortality in low-and middle-income countries: a systematic review protocol

Journal:	*BMJ Open*
Manuscript ID	bmjopen-2021-051792.R1
Article Type:	Protocol
Date Submitted by the Author:	20-Oct-2021
Complete List of Authors:	Griswold, Dylan; Cambridge University, Clinical Neurosciences; Stanford University School of Medicine<br>RUBIANO, ANDRES M; Università degli Studi del Piemonte Orientale Amedeo Avogadro Scuola di Medicina; Meditech Foundation
**Primary Subject Heading**:	Health services research
Secondary Subject Heading:	Global health
Keywords:	ACCIDENT & EMERGENCY MEDICINE, TRAUMA MANAGEMENT, Health policy < HEALTH SERVICES ADMINISTRATION & MANAGEMENT

SCHOLARONE™
Manuscripts

The role of mHealth applications for Emergency Medical System activation in reducing mortality in low-and middle-income countries: a systematic review protocol

Authors

Dylan P Griswold, BA[1,2,3] dg550@cam.ac.uk **Andres M. Rubiano, MD, PhD(c)**[1,4,5] andresrubiano@aol.com

1 NIHR Global Health Research Group on Neurotrauma, University of Cambridge, Cambridge, UK 2 Division of Neurosurgery, Department of Clinical Neurosciences, Addenbrooke's Hospital & University of Cambridge, Cambridge, UK 3 Stanford University School of Medicine, Stanford, CA, USA 4 Università del Piemonte Orientale (UPO) and Vrije Universiteit Brussel (VUB). 5 Meditech Foundation, Cali, Colombia.

Correspondence:

Andres M. Rubiano

Neuroscience Institute, INUB-MEDITECH Research Group

El Bosque University

Bogotá, Colombia

andresrubiano@aol.com

+57 300 6154775

For peer review only

Abstract

Introduction: Prehospital care is an essential component in reducing mortality for patients presenting with emergency medical conditions. Prehospital systems tend to be underdeveloped or non-existent in these areas, with less than 1% of LMIC populations served by an organized prehospital system. Mobile health apps for activation of Emergency Medical System (EMS) have been shown to decrease mortality, but there has yet to be a systematic review and meta-analysis performed to clarify the role that these apps play in reducing mortality in LMICs. The objective of this review is to evaluate the effectiveness of mobile health apps for EMS activation versus traditional EMS dispatches in survival and transport time in patients with emergency medical conditions.

Methods and Analysis: The proposed systematic review of randomized controlled trials (RCTs) and non-randomized controlled trials (NRCTs) will be conducted in accordance with the Joanna Briggs Institute methodology for systematic reviews of effectiveness evidence. MEDLINE, CINAHL, Web of Science, Cochrane Library, EMBASE, and EBSCO will be searched from January 2005 to March 2021. The search results will be presented according to the Preferred Reporting Items for Systematic Reviews and Meta-Analyses (PRISMA) flow diagram. Primary outcomes will include mortality and transport time. Critical appraisal will be assessed using the JBI SUMARI Risk-of-Bias Tool for RCTs, and Risk-of-Bias In Non-Randomized Studies tool for NRCTs. A narrative synthesis will be conducted for all included studies. If sufficient data are available, a meta-analysis will be conducted. I^2 statistics will be used to assess heterogeneity and identify their potential sources.

Ethics and Dissemination: No ethical approval will be required, as this review is based on already published data and does not involve interaction with human subjects. The plan for dissemination, however, is to publish the findings of the review in a peer-reviewed journal and present findings at high- level international conferences that engage the most pertinent stakeholders. Any amendments to this protocol will be documented in the final review.

Prospero Registration: CRD42021243041

For peer review only

ARTICLE SUMMARY

Strengths and Limitations of this study

- To the best of our knowledge this protocol provides a detailed description of the first systematic review on the effectiveness of mobile health apps for EMS activations in reducing mortality in low-and middle-income countries
- The protocol adheres to the Preferred Reporting Items for Systematic Reviews and Meta-Analyses Protocols guidelines for reporting a systematic review protocol
- Given the niche nature of the topic it is possible there may not be enough data to perform a meta-analysis

For peer review only

Introduction

Prehospital care is an essential component in reducing mortality for patients presenting with emergency medical conditions. Prehospital systems tend to be underdeveloped or non-existent in these areas, with less than 1% of low-and middle-income country (LMIC) populations served by an organized prehospital system.[1] Mobile health apps for Emergency Medical Systems (EMS) activation have been shown to decrease mortality, but EMS development in many LMICs is in its infancy and remains underdeveloped compared to the already existing need for better prehospital care and transport. [2–5] A literature review evaluating the status of EMS in 16 LMICs found that most of the included countries lacked an organized prehospital system and were greatly hindered by financial constraints.[6] Another constraint was found to be a lack of the importance of an EMS among locals. The review found that countries with well-developed prehospital care like the US and the UK had the most influence in the development of well-functioning EMS through the implementation of training programs, support of local EMS leadership, advocacy and awareness, and funding.

Mobile health (mHealth) services hold promises in helping bridge the gap between the organization of a functional EMS and a well-functioning prehospital system. There is a wide range of cheap, easily accessible tools which can be used at the point of dispatch or care delivery. With a rapid increase in cellular services, health workers have new opportunities to reach and treat patients that they could not before. There has been an increase in the number of mHealth projects currently active in LMICs, but these tend to be pilot studies that have not been scaled up.[7] The good news is that with COVID19 came a surge in implementation of mHealth strategies to improve pandemic management and response. Countries in which these strategies were used for planning, surveillance, testing, contact tracing, quarantine, and healthcare were more successful.[8] Countries that can leverage this existing network to improve their EMS will likely find a noticeable improvement in prehospital care soon.

While it is unlikely there is a large body of studies examining the effect of mHealth apps for EMS activation regarding mortality and transport time in LMIC populations, it is important to gain a better understanding of the studies that have been published and for which there is data worth appraising. A systematic review and meta-analysis to clarify the role mHealth apps for EMS activation play in reducing mortality in LMICs has not yet been written. Our objective is to carry this out to provide data that will help improve mHealth apps for EMS dispatch in LMICs.

Methods

A protocol of this systematic review following the PRISMA statement was registered in the International Prospective Registry of Systematic Reviews. Any changes to the protocol will be amended in PROSPERO and reported in the final review. This systematic review was conducted following the JBI methodology for systematic reviews and meta-analyses.[9,10] This protocol adheres to the Preferred Reporting Items for Systematic Reviews and Meta-Analyses protocols (PRISMA-P) 2015.[10,11]

Patient and public involvement

Patients and the public were not involved in the design of this systemic review protocol.

Study design

For peer review only

A systematic review of peer review literature following the Preferred Reporting Items for Systematic Reviews and Meta-Analyses (PRISMA) approach by Moher et al is planned for this review.[10,11] Figure 1 summarises the planned stages of the review as described in this protocol.

Data source and search strategy

The search strategy will aim to locate both published and unpublished studies, including the grey literature. An initial limited search of MEDLINE (via PubMed) and CINAHL was undertaken to identify articles on the topic. The text words contained in the titles and abstracts of relevant articles, and the index terms used to describe the articles will be used to develop a full search strategy for Web of Science, Cochrane Library, EMBASE, and CINAL (EBSCO) will be searched from January 2005 to March 2021 to reflect contemporary practice *(Supplement 1*). There will be no restriction regarding the language. The search strategy, including all identified keywords and index terms, will be adapted for each included database and/or information source. The reference list of all included sources of evidence will be screened for additional studies. EndNote20 will be used to store, organize, and manage all references

The existence of controlled descriptors (MeSH terms and CINAHL headings) and their key words was verified in each database. The search terms were combined using the Boolean operators 'AND' and 'OR.'[12]

A search strategy combining MeSH terms and free-text words such as –(mobile OR telemedicine* OR ehealth OR telehealth OR smartphone* OR "smart phone*") AND ("emergency medical service*" OR "emergency medical system*" OR "emergency health service*" OR EMS) AND Cochrane's LMIC search filter.[13]

Selection of studies

Following the search, all identified citations will be collated and uploaded into EndNote20 (Clarivate Analytics, PA, USA). The citations will then be imported into JBI SUMARI for the review process. Two independent reviewers will examine titles and abstracts for eligibility. The full text of selected studies will be retrieved and assessed. Full-text studies that do not meet the inclusion criteria will be excluded, and a list of such excluded studies will be provided. Disagreements between the reviewers during title and abstract screening or full-text screening, will be resolved by consensus, or with a third reviewer. The results of the search will be reported in full in the final report and presented in a Preferred Reporting Items for Systematic Reviews and Meta-analyses (PRISMA) flow diagram.

Eligibility criteria

Inclusion criteria

Participants

This review will include patients of any age with emergency medical conditions including but not limited to stroke, myocardial infarction, trauma, and respiratory failure.

Intervention(s)

For peer review only - http://bmjopen.bmj.com/site/about/guidelines.xhtml

Mobile health EMS dispatch, alerts, triage systems and applications

Comparator(s)

Traditional EMS dispatch, alerts, and triage systems.

Outcomes

This review will consider studies that include the following outcomes: mortality and transport time expressed as incidence differences, risk ratios or odds ratios. All outcomes will be summarized narratively.

Types of studies

This review will consider both experimental and quasi-experimental study designs including randomized controlled trials, non-randomized controlled trials, before and after studies and interrupted time-series studies. In addition, analytical observational studies including prospective and retrospective cohort studies, case-control studies and analytical cross-sectional studies will be considered for inclusion. This review will also consider descriptive observational study designs including case series, individual case reports and descriptive cross-sectional studies for inclusion. We will include preprint studies identified in our search, but no ongoing studies will be considered.

Assessment of methodological quality

Eligible studies will be critically appraised by two independent reviewers at the study level using standardized critical appraisal instruments from the Joanna Briggs Institute for experimental and quasi-experimental studies. Authors of papers will be contacted to request missing or additional data for clarification, where required. Any disagreements that arise will be resolved through discussion, or with a third reviewer. The results of critical appraisal will be reported in narrative form and in a table.

All studies, regardless of the results of their methodological quality, will undergo data extraction and synthesis (where possible).

Data extraction

Data will be extracted from studies included in the review by two independent reviewers using the standardized data extraction tool. The data extracted will include specific details about the populations, study methods, interventions, and outcomes of significance to the review objective. Any disagreements that arise between the reviewers will be resolved through discussion, or with a third reviewer. Authors of papers will be contacted to request missing or additional data, where required.

Data synthesis

Studies will, where possible be pooled in statistical meta-analysis using JBI SUMARI. Effect sizes will be expressed as either odds ratios (for dichotomous data) and weighted (or standardized) final post-intervention mean differences (for continuous data) and their 95% confidence intervals will be calculated for analysis. Heterogeneity will be assessed statistically using the standard chi squared and I^2 tests. Statistical analyses will be determined by the final heterogeneity in the included studies.[13]

For peer review only

Sensitivity analyses will also be conducted. Where statistical pooling is not possible the findings will be presented in narrative form including tables and figures to aid in data presentation where appropriate. A funnel plot will be generated in Microsoft Excel (Seattle, WA) to use to assess publication bias if there are 10 or more studies included in a meta-analysis. Statistical tests for funnel plot asymmetry (Egger test, Begg test, Harbord test) will be performed where appropriate.

Assessing certainty in the findings

The Grading of Recommendations, Assessment, Development and Evaluation (GRADE) approach for grading the certainty of evidence will be followed.[14] The SoF will present the following information where appropriate: absolute risks for the treatment and control, estimates of relative risk, and quality of the evidence based on the risk of bias, directness, heterogeneity, precision, and risk of publication bias. The outcomes reported in the SoF will be mortality and transport time.

Ethics and dissemination

No ethical approval will be required, as this review is based on already published data and does not involve interaction with human subjects. The plan for dissemination, however, is to publish the findings of the review in a peer-reviewed journal and present findings at high- level international conferences that engage the most pertinent stakeholders. Any amendments to this protocol will be documented in the final review.

DISCUSSION

This protocol has been rigorously developed and designed specifically to assess the effects of mHealth apps for EMS activation on mortality and transport time in LMICs. Given the likely limited evidence associated with the primary objective, findings from the review will be critical for researchers, policymakers, government, and non-governmental organisations for planning, developing, and improving mHealth apps for EMS activation in LMICs. If protocol modifications are required, the authors will include the detailed description of any changes along with a justification during the publication of the review.

The main barriers for implementing mHealth apps for EMS activation include regulatory bodies, technological expertise, and user interaction. Usability of the application, signal loss, data volume utilization, need to enter passwords, and the availability of automated or in-app context-relevant clinical advice are important considerations for the three groups involved: experts, front-line healthcare workers, and patients. This review will serve as an important role as a repository of available evidence for the purpose of addressing these barriers and setting effective policy and guideline recommendations.

For peer review only

Contributors AMR conceived the review. DG wrote an initial draft of the manuscript. DG and AMR refined the review design. Both authors were involved in subsequent draft manuscript reviews and updates and approved the final version of this protocol.

Funding Mr Griswold was supported by the Gates Cambridge (Grant #: OPP1144) and Dr Rubiano was supported by the CRIMEDIM center at Università del Piemonte Orientale (UPO) (Grant #: N/A).

Competing interests None declared.

Patient consent for publication Not required.

Data statement: This review is based on previously published data. Any relevant data will be published with the review as either an appendix or as an online supplement.

Acknowledgements: This article is the result of a study conducted in the framework of the International PhD in Global Health, Humanitarian Aid and Disaster Medicine jointly organized by Università del Piemonte Orientale (UPO) and Vrije Universiteit Brussel (VUB). The authors would like to thank Stanford University research librarian, Lily Ren for her invaluable contribution in helping to develop a relevant and robust search strategy.

Open access This is an open access article distributed in accordance with the Creative Commons Attribution Non-Commercial (CC BY-NC 4.0) license, which permits others to distribute, remix, adapt, build upon this work non-commercially, and license their derivative works on different terms, provided the original work is properly cited, appropriate credit is given, any changes made indicated, and the use is non-commercial. See: http://creativecommons.org/licenses/by-nc/4.0/.

License to BMJ Group for Publication: I ***Andres M. Rubiano*** The Corresponding Author of this article contained within the original manuscript which includes any diagrams & photographs within and any related or stand-alone film submitted (the Contribution") has the right to grant on behalf of all authors and does grant on behalf of all authors, a license to the BMJ Publishing Group Ltd and its licensees, to permit this Contribution (if accepted) to be published in the BMJ and any other BMJ Group products and to exploit all subsidiary rights, as set out in our license set out at: http://www.bmj.com/about-bmj/resources-authors/forms-policies-and-checklists/copyright-open-access-and-permission-reuse.

For peer review only - http://bmjopen.bmj.com/site/about/guidelines.xhtml

References

1. Nielsen K, Mock C, Joshipura M, Rubiano AM, Zakariah A, Rivara F. Assessment of the Status of Prehospital Care in 13 Low- and Middle-Income Countries. *Prehosp Emerg Care*. 2012;16(3):381–9.

2. Mbugua P, Otieno C, Kayima J, Amayo A, McLigeyo S. Diabetic ketoacidosis: clinical presentation and precipitating factors at Kenyatta National Hospital, Nairobi. *E Afr Med J*. 2006;82(12):S191-6.

3. Gaieski DF, Goyal M. History and current trends in sudden cardiac arrest and resuscitation in adults. *Hosp Pract 1995*. 2010;38(4):44–53.

4. Terkelsen CJ, Sørensen JT, Maeng M, Jensen LO, Tilsted H-H, Trautner S, Vach W, Johnsen SP, Thuesen L, Lassen JF. System Delay and Mortality Among Patients With STEMI Treated With Primary Percutaneous Coronary Intervention. *Jama*. 2010;304(7):763.

5. Siman-Tov M, Radomislensky I, ITG, Peleg K. Reduction in trauma mortality in Israel during the last decade (2000–2010): The impact of changes in the trauma system. *Inj*. 2013;44(11):1448–52.

6. Suryanto, Plummer V, Boyle M. EMS Systems in Lower-Middle Income Countries: A Literature Review. *Prehospital Disaster Medicine*. 2016;32(1):64–70.

7. Wallis L, Blessing P, Dalwai M, Shin SD. Integrating mHealth at point of care in low- and middle-income settings: the system perspective. *Global Health Action*. 2017;10(sup3):1327686.

8. Whitelaw S, Mamas MA, Topol E, Spall HGCV. Applications of digital technology in COVID-19 pandemic planning and response. *Lancet Digital Heal*. 2020;2(8):e435–40.

9. Aromataris E, Munn Z, Tufanaru C, Campbell J, Hopp L. JBI Reviewer's Manual. 2019;

10. Moher D, Liberati A, Tetzlaff J, Altman DG, Group P. Preferred reporting items for systematic reviews and meta-analyses: the PRISMA statement. *International journal of surgery (London, England)*. 2010;8:336–41.

11. Lefebvre C, Manheimer E, Glanville J. Cochrane Handbook for Systematic Reviews of Interventions. 2008;95–150.

12. Cochrane, Library W, Collaboration C. Cochrane LMIC Filters [Internet]. undefined. 2020 [cited 2021 Mar 26]. Available from: https://epoc.cochrane.org/lmic-filters

13. Tufanaru C, Munn Z, Stephenson M, Aromataris E. Fixed or random effects meta-analysis? Common methodological issues in systematic reviews of effectiveness. *Int J Evidence-based Healthc*. 2015;13(3):196–207.

14. Grading quality of evidence and strength of recommendations. *Bmj*. 2004;328(7454):1490.

Figure 1. Summary of search strategy process

For peer review only

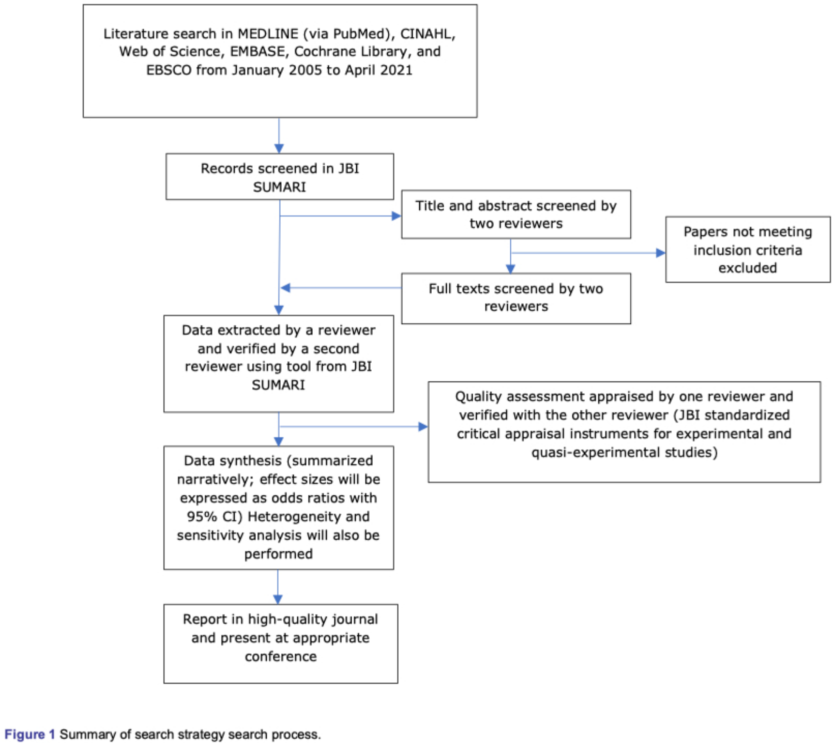

Figure 1. Summary of search strategy process

340x288mm (72 x 72 DPI)

Supplement 1
MEDLINE via PubMed search strategy

Search conducted on 26 March 2021

Search	Query	Results
#12	Search: **#11 AND #10** Sort by: **Most Recent**	397
#11	Search: **("Smartphone"[Mesh] OR "Telemedicine"[Mesh] OR "Mobile Applications"[Mesh] OR "mobile"[tw] OR "ehealth"[tw] OR "telehealth"[tw] OR "telemedicine"[tw] OR "smartphone*"[tw] OR "smart phone*"[tw]) AND ("Emergency Medical Services"[Mesh] OR "emergency medical service*"[tw] OR "emergency medical system*"[tw] OR "emergency health service*"[tw] OR EMS[tw])** Sort by: **Most Recent**	4,072
#10	Search: **afghanistan[MeSH] OR albania[MeSH] OR algeria[MeSH] OR american samoa[MeSH] OR angola[MeSH] OR antigua and barbuda[MeSH] OR argentina[MeSH] OR armenia[MeSH] OR aruba[MeSH] OR azerbaijan[MeSH] OR bahrain[MeSH] OR bangladesh[MeSH] OR barbados[MeSH] OR republic of belarus[MeSH] OR belize[MeSH] OR benin[MeSH] OR bhutan[MeSH] OR bolivia[MeSH] OR bosnia and herzegovina[MeSH] OR botswana[MeSH] OR brazil[MeSH] OR bulgaria[MeSH] OR burkina faso[MeSH] OR burundi[MeSH] OR cabo verde[MeSH] OR cambodia[MeSH] OR cameroon[MeSH] OR central african republic[MeSH] OR chad[MeSH] OR chile[MeSH] OR china[MeSH] OR colombia[MeSH] OR comoros[MeSH] OR democratic republic of the congo[MeSH] OR congo[MeSH] OR costa rica[MeSH] OR cote d'ivoire[MeSH] OR croatia[MeSH] OR cuba[MeSH] OR cyprus[MeSH] OR czech republic[MeSH] OR djibouti[MeSH] OR dominica[MeSH] OR dominican republic[MeSH] OR ecuador[MeSH] OR egypt[MeSH] OR el salvador[MeSH] OR equatorial guinea[MeSH] OR eritrea[MeSH] OR estonia[MeSH] OR swaziland[MeSH] OR ethiopia[MeSH] OR fiji[MeSH] OR gabon[MeSH] OR gambia[MeSH] OR ghana[MeSH] OR gibraltar[MeSH] OR greece[MeSH] OR grenada[MeSH] OR guam[MeSH] OR guatemala[MeSH] OR guinea[MeSH] OR guinea bissau[MeSH] OR guyana[MeSH] OR haiti[MeSH] OR honduras[MeSH] OR hungary[MeSH] OR india[MeSH] OR indonesia[MeSH] OR iran[MeSH] OR iraq[MeSH] OR jamaica[MeSH] OR jordan[MeSH] OR kazakhstan[MeSH] OR kenya[MeSH] OR democratic people's republic of korea[MeSH] OR republic of korea[MeSH] OR kosovo[MeSH] OR kyrgyzstan[MeSH] OR laos[MeSH] OR latvia[MeSH] OR lebanon[MeSH] OR lesotho[MeSH] OR liberia[MeSH] OR libya[MeSH] OR lithuania[MeSH] OR macau[MeSH] OR republic of north macedonia[MeSH] OR madagascar[MeSH] OR malawi[MeSH] OR malaysia[MeSH] OR indian ocean islands[MeSH] OR mali[MeSH] OR malta[MeSH] OR micronesia[MeSH] OR palau[MeSH] OR mauritania[MeSH] OR mauritius[MeSH] OR mexico[MeSH] OR moldova[MeSH] OR mongolia[MeSH] OR montenegro[MeSH] OR morocco[MeSH] OR mozambique[MeSH] OR myanmar[MeSH] OR namibia[MeSH] OR nepal[MeSH] OR netherlands antilles[MeSH] OR nicaragua[MeSH] OR niger[MeSH] OR nigeria[MeSH] OR oman[MeSH] OR pakistan[MeSH] OR panama[MeSH] OR papua new guinea[MeSH] OR paraguay[MeSH] OR peru[MeSH] OR philippines[MeSH] OR poland[MeSH] OR portugal[MeSH] OR puerto rico[MeSH] OR romania[MeSH] OR russia[MeSH] OR rwanda[MeSH] OR samoa[MeSH] OR sao tome and principe[MeSH] OR saudi arabia[MeSH] OR senegal[MeSH] OR serbia[MeSH] OR seychelles[MeSH] OR sierra leone[MeSH] OR slovakia[MeSH] OR slovenia[MeSH] OR melanesia[MeSH] OR somalia[MeSH] OR south africa[MeSH] OR south sudan[MeSH] OR sri lanka[MeSH] OR saint kitts and nevis[MeSH] OR saint lucia[MeSH] OR saint vincent and the grenadines[MeSH] OR sudan[MeSH] OR suriname[MeSH] OR syria[MeSH] OR tajikistan[MeSH] OR tanzania[MeSH] OR thailand[MeSH] OR timor leste[MeSH] OR togo[MeSH] OR tonga[MeSH] OR trinidad and tobago[MeSH] OR tunisia[MeSH] OR turkey[MeSH] OR turkmenistan[MeSH] OR uganda[MeSH] OR ukraine[MeSH] OR uruguay[MeSH] OR uzbekistan[MeSH] OR vanuatu[MeSH] OR venezuela[MeSH] OR vietnam[MeSH] OR middle east[MeSH] OR yemen[MeSH] OR yugoslavia[MeSH] OR zambia[MeSH] OR zimbabwe[MeSH] OR africa south of the sahara[MeSH] OR africa, central[MeSH] OR africa, northern[MeSH] OR africa, southern[MeSH] OR africa, eastern[MeSH] OR africa, western[MeSH] OR west indies[MeSH] OR indian ocean islands[MeSH] OR caribbean region[MeSH] OR central america[MeSH] OR latin america[MeSH] OR south america[MeSH] OR asia, central[MeSH] OR asia, northern[MeSH] OR asia, southeastern[MeSH] OR asia, western[MeSH] OR europe, eastern[MeSH] OR developing countries[MeSH]** Sort by: **Most Recent**	1,473,497

ew only

PRISMA-P 2015 Checklist

This checklist has been adapted for use with protocol submissions to *Systematic Reviews* from Table 3 in Moher D et al: Preferred reporting items for systematic review and meta-analysis protocols (PRISMA-P) 2015 statement. *Systematic Reviews* 2015 **4**:1

Section/topic	#	Checklist item	Information reported Yes	Information reported No	Line number(s)
**ADMINISTRATIVE INFORMATION**					
**Title**					
Identification	1a	Identify the report as a protocol of a systematic review	☒	☐	2,3
Update	1b	If the protocol is for an update of a previous systematic review, identify as such	☐	☒	
**Registration**	2	If registered, provide the name of the registry (e.g., PROSPERO) and registration number in the Abstract	☐	☒	
**Authors**					
Contact	3a	Provide name, institutional affiliation, and e-mail address of all protocol authors; provide physical mailing address of corresponding author	☒	☐	5-9
Contributions	3b	Describe contributions of protocol authors and identify the guarantor of the review	☒	☐	193-195
**Amendments**	4	If the protocol represents an amendment of a previously completed or published protocol, identify as such and list changes; otherwise, state plan for documenting important protocol amendments	☐	☒	
**Support**					
Sources	5a	Indicate sources of financial or other support for the review	☒	☐	196-197
Sponsor	5b	Provide name for the review funder and/or sponsor	☒	☐	196-197
Role of sponsor/funder	5c	Describe roles of funder(s), sponsor(s), and/or institution(s), if any, in developing the protocol	☒	☐	196-197
**INTRODUCTION**					
**Rationale**	6	Describe the rationale for the review in the context of what is already known	☒	☐	45-68
**Objectives**	7	Provide an explicit statement of the question(s) the review will address with reference to participants, interventions, comparators, and outcomes (PICO)	☒	☐	69-74
**METHODS**					

For peer review only

BioMed Central
The Open Access Publisher

Section/topic	#	Checklist item	Information reported		Line number(s)
			Yes	No	
**Eligibility criteria**	8	Specify the study characteristics (e.g., PICO, study design, setting, time frame) and report characteristics (e.g., years considered, language, publication status) to be used as criteria for eligibility for the review	X		115-135
**Information sources**	9	Describe all intended information sources (e.g., electronic databases, contact with study authors, trial registers, or other grey literature sources) with planned dates of coverage	X		89-98
**Search strategy**	10	Present draft of search strategy to be used for at least one electronic database, including planned limits, such that it could be repeated	X		234…
STUDY RECORDS					
Data management	11a	Describe the mechanism(s) that will be used to manage records and data throughout the review	X		107-108
Selection process	11b	State the process that will be used for selecting studies (e.g., two independent reviewers) through each phase of the review (i.e., screening, eligibility, and inclusion in meta-analysis)	X		109-114
Data collection process	11c	Describe planned method of extracting data from reports (e.g., piloting forms, done independently, in duplicate), any processes for obtaining and confirming data from investigators	X		145-149
**Data items**	12	List and define all variables for which data will be sought (e.g., PICO items, funding sources), any pre-planned data assumptions and simplifications		X	
**Outcomes and prioritization**	13	List and define all outcomes for which data will be sought, including prioritization of main and additional outcomes, with rationale	X		146-149
**Risk of bias in individual studies**	14	Describe anticipated methods for assessing risk of bias of individual studies, including whether this will be done at the outcome or study level, or both; state how this information will be used in data synthesis	X		137-143
DATA					
**Synthesis**	15a	Describe criteria under which study data will be quantitatively synthesized		X	
	15b	If data are appropriate for quantitative synthesis, describe planned summary measures, methods of handling data, and methods of combining data from studies, including any planned exploration of consistency (e.g., I^2, Kendall's tau)	X		151-155
	15c	Describe any proposed additional analyses (e.g., sensitivity or subgroup analyses, meta-regression)	X		155-157
	15d	If quantitative synthesis is not appropriate, describe the type of summary planned		X	
**Meta-bias(es)**	16	Specify any planned assessment of meta-bias(es) (e.g., publication bias across studies, selective reporting within studies)	X		157-160
**Confidence in cumulative evidence**	17	Describe how the strength of the body of evidence will be assessed (e.g., GRADE)	X		162-166
